# Organizational culture in the primary healthcare setting of Cyprus

**DOI:** 10.1186/1472-6963-13-112

**Published:** 2013-03-24

**Authors:** Theodora Zachariadou, Savvas Zannetos, Andreas Pavlakis

**Affiliations:** 1 Open University of Cyprus, Nicosia, Cyprus; 2 Planning Bureau, Open University of Cyprus, Nicosia, Cyprus

**Keywords:** Cyprus, Primary healthcare, Organizational, Culture

## Abstract

**Background:**

The concept of organizational culture is important in understanding the behaviour of individuals in organizations as they manage external demands and internal social changes. Cyprus healthcare system is under restructuring and soon a new healthcare scheme will be implemented starting at the Primary Healthcare (PHC) level. The aim of the study was to investigate the underlying culture encountered in the PHC setting of Cyprus and to identify possible differences in desired and prevailing cultures among healthcare professionals.

**Methods:**

The population of the study included all general practitioners (GPs) and nursing staff working at the 42 PHC centres throughout the island. The shortened version of the Organizational Culture Profile questionnaire comprising 28 statements on organizational values was used in the study. The instrument was already translated and validated in Greek and cross-cultural adaptation was performed. Participants were required to indicate the organization’s characteristic cultural values orientation along a five-point Likert scale ranging from “Very Much = 1” to “Not at all= 5”. Statistical analysis was performed using SPSS 16.0. Student t-test was used to compare means between two groups of variables whereas for more than two groups analysis of variance (ANOVA) was applied.

**Results:**

From the total of 306 healthcare professionals, 223 participated in the study (72.9%). The majority of participants were women (75.3%) and mean age was 42.6 ± 10.7 years. Culture dimension “performance orientation” was the desired culture among healthcare professionals (mean: 1.39 ± 0.45). “Supportiveness” and “social responsibility” were the main cultures encountered in PHC (means: 2.37 ± 0.80, 2.38 ± 0.83). Statistical significant differences were identified between desired and prevailing cultures for all culture dimensions (p= 0.000).

**Conclusions:**

This was the first study performed in Cyprus assessing organizational culture in the PHC setting. In the forthcoming health system reform, healthcare professionals will face challenges both at organizational level and professional status. Results of the study can serve as background knowledge for leaders and policy makers who seek interventions to improve performance before the implementation of a new national healthcare scheme.

## Background

The concept of organizational culture emerges from various disciplines including anthropology, sociology, and management [1]. Recent interest in the culture of healthcare organizations has begun to address the importance of culture for key organizational outcomes [2]. For example, healthcare cultures that emphasize group affiliation, teamwork and coordination have been associated with greater implementation of quality improvement practices [3]. According to Rundall et al. [4], the culture of physician organizations is important in the care of chronic illnesses, as it may relate to the ability of these organizations to support quality improvement efforts and develop information systems to provide better patient care [4]. Other authors have investigated how employee attitudes and behaviors are influenced by the “fit” or congruence between the organization’s culture and the individual’s own values or beliefs about what the organization’s values should be. Different studies have found that greater congruence is associated with more positive employee attitudes [5-9].

Several definitions of organizational culture can be found in the literature. According to O’Reilly and Chatman [10] “culture is a system of shared values defining what is important, and norms defining attitudes and behaviors that guide member’s attitudes and behaviors”. As Schein [11] proposed: “Organizational culture is the pattern of shared basic assumptions - invented, discovered or developed by a given group as it learns to cope with its problems of external adaptation and internal integration - that has worked well enough to be considered valid and therefore to be taught to new members as the correct way to perceive, think and feel in relationship to those problems” [11].

There are a variety of qualitative and quantitative approaches that measure organizational culture depending on the purpose and goals of each investigator [12]. Among the most widely used instruments is the Organizational Culture Inventory [13,14] which evaluates styles within organizational groups to characterize the overall organizational culture along three dimensions (constructive, passive/defensive, or aggressive/defensive orientations) [13]. It comprises 120 items; each one rated on a 1–5 Likert scale [15] and has internal reliability that ranges from 0.67 to 0.92 [16]. Another two questionnaires are designed to measure the culture and values at the organizational level. The first is the Organizational Beliefs Questionnaire [17]. This tool is a 50 item questionnaire that uses a 5-point Likert scale (strongly agree to strongly disagree) and has ten subscales: work should be fun, being the best, innovation, attention to detail, worth and value of people, quality, communicating to get the job done, growth/profit/other indications of success, hand-on management and the importance of a shared philosophy^17^. It has moderate to high internal validity (0.35 to 0.78) [18].

The second tool is the Organizational Culture Profile (OCP), developed by O’Reilly, Chatman and Caldwell [7]. The original version of the OCP consists of 54 value statements with average reliability coefficient of 0.88 [7]. A Q-sort methodology of data collection [19] is used to identify values that characterize a target organization and an individual’s preference for that particular set of values by assessing person- organization fit [7,20]. Based on a review performed by Ashkanasy et al. [21] concerning 18 culture measures published between 1975 and 1992, OCP was found to be one of only a few instruments to provide details concerning reliability and validity. The OCP has since been revised and shortened by Cable and Judge [22] to measure organizational and personal culture orientations. The instrument was further modified by Sarros et al. [23] along with the development of a five point Likert type scale format [15]. The new shortened version of the OCP consists of a 28-item, seven factor structure comprising the following factors: supportiveness, innovation, competitiveness, performance orientation, stability, emphasis on rewards and social responsibility [23].

### Organization and provision of primary healthcare services in Cyprus

The existing National Healthcare System (NHS) of Cyprus provides almost universal coverage, as 75% of the population has access to healthcare services provided in the public sector. Primary Healthcare (PHC) is usually the first point of contact and is delivered through 42 PHC centers throughout the island. Fourteen of those are located in urban areas, 22 are rural and six are part of the outpatient department clinics of each town’s general hospital. The personnel at each PHC center comprise general practitioners (GPs), dentists, psychiatrists, pharmacists, nursing and administrative staff [24].

In the present NHS, the Ministry of Health is responsible for the organization and delivery of primary, secondary and tertiary healthcare. Human resource management at the public healthcare sector is highly centralized and bureaucratic. Consequently, recruitment and selection of employees is controlled by the central authorities who are unable to ensure person-organization fit, motivation or reward systems. Rural PHC centers are mostly understaffed whereas urban PHC centers have an excess in technical and human resources leading to inequalities in the care provided [25].

Cyprus healthcare system is passing through a period of transformation. Since 2001, the Ministry of Health has been working on a comprehensive health care reform. A new national health scheme that strengthens the role of PHC was approved by law a decade ago [26]. According to the new scheme [26], PHC will become the cornerstone of an effective healthcare system in which GPs will act as gatekeepers to secondary and tertiary healthcare services and where PHC centers will form independent health units. In the forthcoming implementation of the new health care system, GPs will be assigned with new tasks such as human and financial resource management.

The aim of the present study was: a) to describe the organizational culture pattern in the PHC setting of Cyprus, b) to compare relative strengths of different culture patterns between healthcare professionals and c) to evaluate the “fit” between employees’ desired culture and the real life culture perceived by the employees.

## Methods

### Instrument

The abbreviated form of the Organizational Culture Profile questionnaire (OCP) revised by Sarros et al. [23] was used in the present study. The instrument has a Cronbach’s alpha coefficient of 0.75 and each of the seven composite factors includes four value items [23]. Permission to use was obtained by Professor James Sarros.

The original OCP instrument developed by O’Reilly, Chatman and Caldwell [7] was already translated and validated in Greek by Bellou V [27]. Since Greek is the official language of Cyprus, a cross-cultural adaptation of the questionnaire was decided. For this reason, a pretest study was performed in 20 employees of Nicosia General Hospital. During the pretest study, participants were asked to complete the questionnaire and comment on words or sentences that were difficult to understand as well as to rephrase problematic items [28]. Two statements out of 28 were rephrased in the Greek-Cypriot version of the questionnaire.

A pilot study was then performed in 30 randomly selected registered nurses and specialized physicians of Nicosia General Hospital. Participants were asked to complete the questionnaire at time zero and two weeks later. Test-retest reliability expressed by Pearson correlation coefficient yielded a value of 0.78, whereas internal consistency expressed by Cronbach’s alpha was 0.91. A correlation coefficient of 0.78 indicates a good to excellent reproducibility [29,30] whereas a reliability of 0.91 is considered excellent [31]. In order to examine the nested structure of the data (healthcare professionals working in urban PHC centers/ rural PHC centers/ outpatient departments of the General hospitals of the same healthcare organization) mixed models were applied. Data analysis confirmed that none of the factors used as dependent variables showed statistical significance within each PHC working setting (p value for each factor >0.05). Intra-class correlation coefficient was calculated for participants from each PHC working setting. Relevant values were 0.94 for urban PHC centers, 0.88 for rural PHC centers and 0.90 for outpatient departments, indicating almost perfect agreement (values > 0.80).

Confirmatory factor analysis (CFA) was further applied to the 28 questions to determine whether they could be collapsed into different composite factors in the Cypriot version of the questionnaire. Principal axis factoring extraction with a varimax rotation was performed to analyze the structure of the questionnaire. To establish a composite factor, eigenvalues had to be greater than one and each item had to have a factor loading greater than or equal to 0.5. The suitability of the data for structure detection was examined with Barlett’s Test of Sphericity with p-value of 0.000 and the Kaiser-Meyer-Olkin measure of sampling adequacy measured at 0.846. This procedure resulted in six composite factors. Two items from the factor “innovation” namely risk taking and quick to take advantage of opportunities were loaded to the factor “competitiveness” whereas the other two items (being innovative and taking individual responsibility) were loaded in the factor “performance orientation”. Since the test-retest reliability and internal consistency of the OCP tool were high (0.78 and 0.91 respectively), we decided to use the seven factor structure proposed by Sarros et al. [23]. Composite factors were psychometrically reliable with Cronbach’s α coefficients above the standard of 0.70 proposed by Nunnally [31].

The final version of the questionnaire was then administered to the study population. Respondents were required to indicate the organization’s characteristic cultural values orientation along a five-point Likert scale [15] ranging from “Very Much = 1” to “Not at all= 5”. Additionally the instrument comprised nine items concerning the demographic characteristics of the participants.

### Study population

The study was performed at the PHC setting of Cyprus. At the time of the study a total of 144 GPs and 162 registered nurses were working in the 42 PHC centers throughout the island [24]. Approvals for the study were obtained from both the Director of Medical Services and the Nursing Services Director of the Ministry of Health. The study was also approved by the National Bioethics Committee of Cyprus.

Questionnaires were officially transferred to the entire PHC centers of Cyprus. Instructions were provided in a cover letter signed by the researchers, describing the study objectives, ensuring response confidentiality and stating that participation was voluntary and anonymous. The main investigator gave three phone calls to the physician in charge at each PHC center. The first call was made a day before the questionnaires were sent in order to explain the purpose of the study and to give instructions for questionnaire collection. The second call was made 15 days later as a reminder for questionnaire completion and the final call was performed at the end of study (one month from the first call) in order to ensure that questionnaires were collected and sent according to the instructions. The study was performed during July 2010.

### Statistical analysis

Descriptive statistics were calculated using the statistical software SPSS version 16.0 [32]. Continuous variables were expressed as means (Standard Deviation) and categorical variables as frequencies and percentages. Student t-test was used to compare means between two groups of variables whereas for more than two groups analysis of variance (ANOVA) was applied. Average factor scores on culture dimensions were further compared using Tukey’s test. For the assessment of person to organization fit, paired t- test was applied so as to compare the means of culture dimensions between employees’ desired culture and the existing culture in PHC. P value < 0.05 was considered statistically significant.

## Results

The overall response rate was 72.9% (223 out of 306 questionnaires were returned). A total of 97 out of 144 GPs (67.4%) and 126 out of 162 registered nurses (77.8%) participated in the survey. The majority of the study population were females (75.3%) and mean age was 42.6 (SD=10.7) years. Demographic characteristics of the participants are shown in Table 1. Statistical significant differences were identified between GPs and nurses in regard to mean years at current working position. Average years in current position were 10 (SD= 9.0) for nurses vs 5.8 (SD= 5.9) for physicians, (p=0.000).

**Table 1:** Characteristics of the study population (N= 223)

**Characteristic**	**Ν (%)**
**Gender**	
*Male*	*54 (24.7)*
*Female*	*165 (75.3)*
**Age (years)**	
*≤ 34*	*51 (26.7)*
*35-44*	*48 (25.1)*
*45-54*	*66 (34.6)*
*≥55*	*26 (13.6)*
**Tenure (years)**	
*1-5*	*56 (26.8)*
*6-15*	*54 (25.8)*
*≥ 16*	*99 (47.4)*
**Tenure (years) at current department**	
*1-5*	*107 (52.7)*
*6-15*	*58 (28.6)*
*≥ 16*	*38 (18.7)*
**Family status**	
*Married*	*164 (76.6)*
*Unmarried*	*32 (15.0)*
*Divorced*	*13 (6.1)*
*Widowed*	*5 (2.3)*
**Medical profession**	
*Family Physician*	*97 (43.5)*
*Registered nurse*	*126 (56.5)*
**Professional position**	
Senior medical officer	7 (3.2)
Medical officer	90 (41.5)
Chief nursing officer	10 (4.6)
Senior nursing officer	17 (7.8)
Nursing officer	93 (42.9)
**Primary Healthcare setting**	
*Urban*	*66 (31.7)*
*Rural*	*103 (49.5)*
*Hospital’s Outpatient department*	*39 (18.8)*
**Town of work**	
*Nicosia*	*99 (45.2)*
*Limassol*	*47 (21.5)*
*Paphos*	*30 (13.7)*
*Larnaca*	*23 (10.5)*
*Famagusta*	*20 (9.1)*

Descriptive statistics and reliability coefficients for each dimension of organizational culture are presented in Table 2. As indicated, all dimensions demonstrate good to high reliability using 0.70 as the commonly accepted cutoff criterion [30].

**Table 2:** Descriptive statistics and reliability coefficients for each dimension of the organizational culture (N= 223)

**Culture dimension**^**†**^	**Mean**	**Standard deviation**	α^*^
Supportiveness	1.42	0.51	0.76
Innovation	1.61	0.60	0.71
Competitiveness	1.72	0.54	0.53
Performance orientation	1.39	0.45	0.78
Stability	1.44	0.56	0.69
Emphasis on rewards	1.46	0.61	0.73
Social responsibility	1.50	0.48	0.62
**Total**			**0.91**

^†^1= Very much, 2= Considerably, 3= Moderately, 4= Minimally, 5= Not at all.

α^*^= Cronbach’s alpha.

As shown in Table 2, the desired type of organizational culture among primary healthcare professionals is “performance orientation” (mean: 1.39 ± 0.45) followed by “supportiveness” (mean: 1.42 ± 0.51). The least desired type of culture in PHC is “competitiveness” (mean: 1.72 ± 0.54). Paired t –test revealed statistical significant differences between desired and prevailing cultures for all cultural dimensions. As shown in Table 3, “supportiveness” and “social responsibility” are the main cultures encountered in the PHC setting of Cyprus (means: 2.37 ± 0.80, 2.38 ± 0.83).

**Table 3:** Differences between desired and existing organizational culture dimensions in the primary healthcare setting

**Culture dimension**	**Mean ± S. D.**	**Paired differences† Mean ± S. D.**	**p-value**
Supportiveness (D)	1.42 ± 0.51	−0.94 ± 0.89	**0.000**
Supportiveness (P)	2.37 ± 0.80		
Innovation (D)	1.61 ± 0.60	−0.91 ± 0.92	**0.000**
Innovation (P)	2.52 ± 0.84		
Competitiveness (D)	1.72 ± 0.54	−0.81 ± 0.84	**0.000**
Competitiveness (P)	2.54 ± 0.78		
Performance orientation (D)	1.39 ± 0.45	−1.02 ± 0.93	**0.000**
Performance orientation (P)	2.41 ± 0.84		
Stability (D)	1.44 ± 0.56	−1.14 ± 0.93	**0.000**
Stability (P)	2.58 ± 0.82		
Emphasis on rewards (D)	1.46 ± 0.61	−1.45 ± 1.10	**0.000**
Emphasis on rewards (P)	2.91 ± 0.94		
Social responsibility (D)	1.50 ± 0.48	−0.88 ± 0.90	**0.000**
Social responsibility (P)	2.38 ± 0.83		

^**†**^Paired differences in means ± S. D: Difference in the mean values of each cultural dimension pair (subtraction of the mean value of desired from prevailing culture).

Desired culture (D): “How important is it for this characteristic to be a part of the organization you work for?.

Prevailing culture (P): “To what extent is your organization recognized for its…?”.

p- value: statistical significance <0.05.

### Differences between the study population and dimensions of organizational culture

Women healthcare professionals working in PHC believe that the characteristics that shape the culture “emphasis on rewards” are more important in comparison to their male colleagues, a finding of statistical significance (mean: 1.40 ± 0.53 vs 1.62 ± 0.78, p= 0.036). Tukey post hoc analysis showed that participants between 35–44 years old consider that organizational culture dimension “performance orientation” is more important as compared to those of ≤ 34 years, a finding of statistical significance (mean: 1.27 ± 0.36 vs 1.52 ± 0.53, p= 0.047).

In regard to the population of GPs there were no statistical significant differences between gender, age, tenure and location of practice. On the other hand, female nurses reported that the values included in the cultures: “supportiveness”, “innovation” and “emphasis on rewards” are more important in comparison to their male counterparts. These differences were statistically significant with means for “supportiveness” 1.41 (women) vs 1.79 (men), p= 0.046, for “innovation” 1.55 vs 1.91, p= 0.042 and for “emphasis on rewards” 1.40 vs 1.87, p=0.030. Statistical significant differences were also found for most dimensions of culture between nurses and tenure. Post hoc analysis with Tukey test showed that nurses with professional experience between 6–15 years believe that those values that constitute the cultures: “supportiveness”, “competitiveness”, “performance orientation”, “stability” and “emphasis on rewards” are more important as compared with their colleagues with tenure up to five years. As displayed in Table 4, these results were statistically significant with relevant p values of 0.049, 0.016, 0.036, 0.026 and 0.009 respectively.

**Table 4:** Culture dimensions according to tenure in years for the nursing population (N= 126)

**Culture dimension**	**Mean**	**S. D.**	**p-value**
**Supportiveness**			**0.049**
*1-5 years*	1.58*	*0.70*	
*6-15*	1.30*	*0.36*	
*≥ 16*	*1.53*	*0.56*	
**Innovation**			**0.464**
*1-5 years*	*1.69*	*0.63*	
*6-15*	*1.52*	*0.43*	
*≥ 16*	*1.64*	*0.65*	
**Competitiveness**			**0.016**
*1-5 years*	2.05*	*0.69*	
*6-15*	1.52*	*0.37*	
*≥ 16*	*1.68*	*0.58*	
**Performance orientation**			**0.036**
*1-5 years*	1.66*	*0.56*	
*6-15*	1.30*	*0.39*	
*≥ 16*	*1.34*	*0.44*	
**Stability**			
*1-5 years*	1.72*	*0.78*	**0.026**
*6-15*	1.27*	*0.40*	
*≥ 16*	*1.52*	*0.63*	
**Emphasis on rewards**			**0.009**
*1-5 years*	1.84*	*0.85*	
*6-15*	1.23*	*0.36*	
*≥ 16*	*1.46*	*0.59*	
**Social responsibility**			
*1-5 years*	*1.71*	*0.51*	**0.386**
*6-15*	*1.45*	*0.44*	
*≥ 16*	*1.47*	*0.47*	

*Post hoc analysis revealed statistical significance between those groups.

p- value: statistical significant difference <0.05.

## Discussion

The present study was the first to be performed in Cyprus that measured existing and desired organizational culture between primary healthcare professionals. “Performance orientation” and “supportiveness” were the two most desired types of organizational culture among GPs and nurses who practice at the PHC setting. On the other hand, “supportiveness” and “social responsibility” were the main cultures encountered in PHC whereas the least prevailing culture was “emphasis on rewards”. According to the OCP instrument, a performance orientation culture is shaped by the following characteristics: enthusiasm for the job, results orientation, highly organized employees and high performance expectations [23]. Organizations that have performance oriented culture emphasize achievement, results and action as important values and use systems that reward employees [33]. Although this culture dimension was the desired one for primary healthcare professionals the existing culture in PHC was “supportiveness”. This type of culture however, was the second most desired culture as pointed by the participants. Values that shape this culture dimension include team orientation, people orientation, collaboration and sharing information freely [23]. Organizations with cultures that are collaborative and emphasize cooperation among employees, are team oriented and members tend to have positive relationships with their coworkers and their managers [34]. According to the European definition of general practice/ family medicine the principles of the discipline include team collaboration, person –centered approach, community and individual responsibility [35]. These characteristics are included in the culture dimension “supportiveness” which is the one promoted in the PHC setting of Cyprus.

An important finding of the study was the gap identified between desired and prevailing cultures, yielding statistical significance for all cultural dimensions. As the study showed, there is not one strong culture that characterizes healthcare professionals who practice in PHC. According to Nystrom [36] the strength of an organization’s culture refers to the degree of consensus among members about which values prevail and which dominate in importance. In organizations with strong cultures most employees show consensus with the values of the organization [37] and their organizations in turn provide more meaning, commitment and guidance to their employees [36]. As other studies have shown [36,38] healthcare organizations exhibiting a strong culture achieve desired outcomes such as commitment and satisfaction which in turn often lead to reduced employee turnover and increased job performance. There is convincing evidence that value congruence between individuals and their organizational context is associated with a more positive subjective experience for the person and better performance for the organization [39].

In a similar study performed in five PHC centers in Crete –Greece, the OCI instrument was applied [40]. The results of that study showed that the existing types of culture were aggressive/defensive and passive/defensive. These culture profiles involve expectations for the members of an organization to approach tasks and interaction with other people in powerful ways to promote their status and security [13]. These norms are similar to the values included in the culture dimension “stability” of the OCP tool namely low conflict, security of employment, and stability.

According to the results of three studies performed in the general public hospitals of Cyprus gaps were identified between desired and existing cultures exhibiting statistical significance for every culture dimension [41-43]. In Larnaca hospital [41] desired cultures were “performance orientation” and “stability” with respective means of 1.46 vs 1.47. In Limassol hospital [42] “emphasis on rewards” and “stability” were the most desired cultures (means: 1.42 vs 1.44 respectively), whereas in Paphos hospital [43] “supportiveness” and “stability” were identified as the most desired ones (means: 1.50 vs 1.52). Results emphasize the fact that there is not a unified strong culture among healthcare professionals who work in the public healthcare sector of Cyprus. In all three hospitals “stability” was identified as a desired culture. This type of culture includes characteristics like low conflict, security of employment and calmness. Stable cultures are predictable, rule oriented and bureaucratic and they are usually encountered in public sector institutions. When the environment is stable and certain, these cultures may help the organization to be effective by providing stable and constant levels of output. These cultures however, prevent quick action and may lead to a misfit in a changing and dynamic environment [44]. According to the results of a study performed by Bellou [27] in 20 public Greek hospitals that used the original 54 item OCP instrument [7] the two most prominent characteristics of culture were “aggressiveness” and “supportiveness”, whereas the least prominent ones were “decisiveness” and “team orientation” [27].

Another important finding of our study was that women healthcare professionals working in PHC value more, those characteristics that shape the culture “emphasis on rewards” in comparison to their male counterparts, a finding of statistical significance. There were also statistical significant differences identified between female and male nurses in regard to the importance given to the following cultures: “supportiveness”, “innovation” and “emphasis on rewards” with women valuing these cultures to a greater extent as compared to men (p value < 0.05 for all cultures). A possible explanation could be that men and women undergo a different professional socialization process in which skills and knowledge are acquired. As a result, women have different professional expectations and different values [45]. Studies have shown that women are more people oriented and team oriented whereas men are more task oriented and competitive [46,47].

A noteworthy finding of the study was the difference identified between nurses with professional experience between 6–15 years and those with tenure up to five years in regard to the importance given for the cultures: “supportiveness”, “competitiveness”, “performance orientation”, “stability” and “emphasis on rewards”. Results yielded statistical significance for the aforementioned cultures (p value < 0.05). One possible explanation could be that newcomers in an organization have different values than those who have been in the organization for longer time. Unmet expectations, the feeling of uncertainty in relation to their work and the lack of congruency between their values and the values of the organization are possible reasons that may contribute to the importance attributed in certain culture categories. As studies have shown, environments that send clear messages to newcomers have the potential to change the most strongly held expectations by newcomers. On the other hand, environments in which newcomers receive ambiguous or conflicting messages from insiders will allow newcomers to maintain their initial expectations [48,49].

### Limitations of the study

The present study addressed the population of primary healthcare professionals throughout Cyprus with a high participation rate (72.9%). Therefore there were no limitations regarding the sampling procedure. A possible limitation of the study was the use of the seven factor structure of the questionnaire although CFA identified six factors (the four items of the factor “innovation” collapsed into the factors “competitiveness” and “emphasis on rewards”). This however does not seem to have an impact on the results given the fact that both validity and reliability of the OCP tool were found to be excellent. In addition, the culture dimension “innovation” was not included between desired or encountered types of culture in PHC as noted by healthcare professionals. This finding further supports the comment that the collapse of this factor has not affected the results.

### Implications of the study

This was the first study performed in a PHC setting that used the revised OCP instrument. The study tried to unveil prevailing and desired organizational culture in the PHC setting of Cyprus in the light of the forthcoming healthcare reform. The revised OCP instrument [23] that was used to determine person-organization fit showed lack of congruency between individual and organizational values. Different employees’ perceptions on desired and existing culture dimensions were identified. Moreover, there was lack of a strong unified organizational culture at the PHC level in Cyprus raising issues on job satisfaction and performance. According to Kantek and Baykal [50], if the individual norms and values of the members of an organization accord with the organizational values and norms, the organizational culture is boosted up; otherwise, the organizational culture is weakened and accompanied by a variety of problems such as conflicts, job dissatisfaction and decrease in motivation and performance [50]. It is therefore important for leaders and policy makers to demonstrate insight into the organization’s culture. An awareness of the underlying forces can help them understand personnel behavior, identify necessary organizational changes and help develop the organization to function more efficiently [51]. As Gabriel [8] proposes, leaders should try to understand organizational culture before trying to “fix” it or directly manage it. Otherwise, trying to force employees to accept organizational values might result in the development of reactive subcultures and countercultures [48]. According to Kilmann [1], changes in organizational culture should be managed from a systems perspective, acknowledging the interconnectedness of the parts. Changes in organizational strategy, structure, reward system, reporting and work procedures may therefore have to be implemented to support cultural change [1].

## Conclusions

This was the first study performed in Cyprus that provided insight into the organizational culture encountered in the PHC setting. The study provided evidence that the modified OCP [23] is a reliable and valid tool which can be easily applied in healthcare organizations including PHC settings. The new abbreviated form of the questionnaire that uses a Likert scale provides a more user- friendly approach to investigate organizational culture in large samples [23].

Results of the study can serve as an audit tool for managers and policy makers in the expected implementation of a new NHS. The revised OCP [23] can be used during different stages in the process of the implementation of the new NHS so as to monitor organizational cultural change in conjunction with changes in values and leadership styles. It can also provide the information needed for recruitment and selection of new employees [23] in the new NHS.

Healthcare professionals working in PHC will face changes both at organizational and professional level as new employment relationships will be developed jeopardizing their permanent working status. Results of the study can be used to set targets for organizational cultural change in the new working environment [23]. Whatever interventions will take place policy makers should ensure that important values fostered in PHC are well preserved.

## Abbreviations

OCP: Organizational Culture Profile; PHC: Primary Healthcare; GPs: General Practitioners; NHS: National Healthcare System.

## Competing interest

The authors declare that they have no competing interests.

## Authors’ contributions

TZ participated in the design and coordination of the study and drafted the manuscript. SZ participated in the design of the study, performed the statistical analysis and helped to draft the manuscript. AP conceived of the study and helped to draft the manuscript. All authors read and approved the final manuscript.

## Pre-publication history

The pre-publication history for this paper can be accessed here:

http://www.biomedcentral.com/1472-6963/13/112/prepub

## References

[B1] Thomas C Ward M Chorba C Kumiega A Measuring and interpreting organizational culture JONA 1990 20 17 24 2189967

[B2] Zazzali JL Alexander JA Shortell SM Burns LR Organizational culture and physician satisfaction with dimensions of group practice Health Serv Res 2007 42 1150 1176 10.1111/j.1475-6773.2006.00648.x 17489908 PMC1955261

[B3] Shortell S O’Brien J Carman J Assessing the impact of continuous quality improvement/ total quality management versus implementation Health Serv Res 1995 30 377 401 7782222 PMC1070069

[B4] Rundall T Shortell S Wang MC As good as it gets? Chronic care management in nine leading US physician organizations BMJ 2002 325 958 964 10.1136/bmj.325.7370.958 12399351 PMC1124449

[B5] Koberg C Chusmir L Organizational culture relationships with creativity and other job-related variables J Bus Res 1987 15 397 409 10.1016/0148-2963(87)90009-9

[B6] Shockley-Zalabak P Morley D Adhering to organizational culture Group Organ Stud 1989 14 483 500 10.1177/105960118901400408

[B7] O’Reilly C Chatman J Caldwell D People and organizational culture: a profile comparison approach to assessing person-organization fit Acad Manage J 1991 34 487 516 10.2307/256404

[B8] Vandenberghe C Organizational culture, person-culture fit and turnover: a replication in the health care industry J Organ Behav 1999 20 175 184 10.1002/(SICI)1099-1379(199903)20:2<175::AID-JOB882>3.0.CO;2-E

[B9] Chow C Harrison G McKinnon J Wu A The organizational culture of public accounting firms: evidence from Taiwanese local and U. S. affiliated firms Account Org Soc 2002 27 347 360 10.1016/S0361-3682(01)00033-2

[B10] O'Reilly CA Chatman JA Cummings L, Staw B Culture as social control: Corporations, cults and commitment Research in organizational behavior 1996 Greenwich, CT: JAI Press 18 166

[B11] Schein E Organizational culture and leadership 1985 1 San Fransisco Jossey-Bass ISBN 0875896391

[B12] Scott T Mannion R Davies H Marshall M The quantitative assessment of organizational culture Health Serv Res 2003 38 923 945 10.1111/1475-6773.00154 12822919 PMC1360923

[B13] Cooke R Lafferty J Organizational Culture Inventory 1987 Plymouth, MI: Human Synergistics

[B14] Seago JA Organizational culture in hospitals: issues in measurement J Nurs Meas 1997 5 165 178 9538588

[B15] Likert R A technique for the measurement of attitudes Arch Psychol 1932 140 1 55

[B16] Cooke RA Rousseau DM Behavioral norms and expectations: a quantitative approach to the assessment of organizational culture Group Organ Stud 1988 13 245 273 10.1177/105960118801300302

[B17] Sashkin M Pillars of excellence: Organizational Beliefs Questionnaire 1984 Bryn Mawr PA: Organizational Design and Development

[B18] Xenikou A Furnham A A correlation and factor analytic study of four questionnaire measures of organizational culture Hum Relat 1996 49 349 369 10.1177/001872679604900305

[B19] Block J The Q-sort method in personality assessment and psychiatric research 1978 Palo Alto, CA: Consulting Psychologists Press

[B20] Agle BR Caldwell CB Understanding research on values in business Bus Soc 1999 38 326 387 10.1177/000765039903800305

[B21] Ashkanasy NM Broadfoot LE Falkus S Questionnaire measures of organizational culture Ashkanasy NM, Wilderom CP & Peterson MF 2000 Thousand Oaks CA: Sage Publications 131 146

[B22] Cable DM Judge TA Interviewers’ perceptions of person-organization fit and organizational selection decisions J Appl Psychol 1997 82 546 561 9378683 10.1037/0021-9010.82.4.546

[B23] Sarros JC Gray J Densten IL Cooper B The Organizational Culture Profile revisited: an Australian perspective Aust J Manag 2005 30 159 182 10.1177/031289620503000109

[B24] Annual Report of the Ministry of Health of Cyprus 2009 2010 Ministry of Health 54 56 Available at: http://www.moh.gov.cy/moh/moh.nsf/

[B25] Review of the Health Care System First report of the study for the National Health Insurance Scheme 1991 2 12 Consultancy team

[B26] General system of Health Law N 2001 89 1 3497 11–18

[B27] Bellou V Identifying organizational culture and subcultures within Greek public hospitals J Health Organ Manag 2008 22 496 509 10.1108/14777260810898714 18959301

[B28] Beaton DE Bombardier C Guillemin F Bosi-Ferraz M Guidelines for the process of cross-cultural adaptation of self-report measures Spine 2000 25 3186 3191 10.1097/00007632-200012150-00014 11124735

[B29] Rosner B Fundamentals of Biostatics 1995 Belmont: Duxbury Press

[B30] Fleiss JL The design and analysis of clinical experiments 1986 New York: Wiley

[B31] Nunnally JC Psychometric Theory 1967 New York: McGraw Hill

[B32] Inc SPSS SPSS (release 16.0) statistical software 2008 Chicago: Illinois, SPSS Inc

[B33] Nohria N Joyce W Roberson B What really works Harv Bus Rev 2003 81 42 52 12858710

[B34] Erdogan B Liden RC Kraimer ML Justice and leader member exchange: The moderating role of organizational culture Acad Manage J 2006 49 395 406 10.5465/AMJ.2006.20786086

[B35] Mola E Eiksson T Ortiz Bueno MJ on behalf of WONCA European Council The European Definition Of General Practice/ Family Medicine 2011 WONCA EUROPE [http://www.woncaeurope.org/gp-definitions]

[B36] Nystrom PC Organizational cultures, strategies and commitments in health care organizations Health Care Manage Rev 1993 18 43 49 8444614

[B37] Chatman JA Eunyoung CS Leading by leveraging culture Calif Manage Rev 2003 45 20 34 10.2307/41166186

[B38] Mowdat RT Porter LW Steers RM Employee-organization linkages 1982 New York: Academic Press

[B39] Kristof-Brown A Stevens C Goal congruence in project teams: Does the fit between members’ personal mastery and performance goals matter? J Appl Psychol 2001 86 1083 1095 11768052 10.1037/0021-9010.86.6.1083

[B40] Rovithis M Measurement of organizational culture, role conflict and role ambiguity of personnel of the health centers in the island of Crete 2005 Master’s thesis Retrieved from: http://elocus.lib.uoc.gr/dlib/4/e/b/metadata-dlib-2005rovithis.tkl. Access code: uch.med.msc//2005rovithis

[B41] Trapalis T The influence of organizational culture on role ambiguity, role comflict and satisfaction in Larnaca General Hospital 2012 Master’s thesis Retrieved from: http://kypseli.ouc.ac.cy. Accession order No. 11128/308

[B42] Papadopoulou D Measuring organizational culture in Limassol Hospital 2011 Master’s thesis Retrieved from: http://kypseli.ouc.ac.cy. Accession order No. 11128/133

[B43] Charalampous D Organiztional culture, role ambiguity, role conflict and satisfaction between health professionals at Paphos General Hospital 2012 Master’s thesis Retrieved from: http://kypseli.ouc.ac.cy. Accession order No. 11128/1103

[B44] Westrum R Increasing the number of guards at nuclear power plants. Risk Analysis An International Journal 2004 24 959 961 10.1111/j.0272-4332.2004.00499.x 15357820

[B45] Brief AP Aldag RJ Van Sell M Melone N Anticipatory socialization and role stress among registered nurses J Health Soc Behav 1979 20 161 166 10.2307/2136436 479527

[B46] Betz M O’Connell L Shephard JM Gender differences in proclivity for unethical behavior J Bus Ethics 1989 8 321 324 10.1007/BF00381722

[B47] Kawatra S Krishnan VR Impact of gender and transformational leadership on organizational culture NMIMS Manag Rev 2004 16 1 6

[B48] Gabriel Y The organizational dream world: workplace stories, fantasies and subjectivity Paper presented at the tenth international Aston/ UMIST conference, organization and control of the labor process 1992 Manchester, England: Alston University

[B49] Colella AJ A role for newcomer pre-entry expectations during organizational entry: Expectation effects on job perceptions. Unpublished doctoral dissertation 1989 Columbus: The Ohio State University PS, NON, UNP

[B50] Kantek F Baykal U Organizational culture in nursing schools in Turkey: faculty members’ perspectives Int Nurs Rev 2009 56 306 312 10.1111/j.1466-7657.2009.00721.x 19702803

[B51] Kilmann RH Beyond the quick fix 1984 San Francisco: Jossey-Bass

